# The social relevance and the temporal constraints of motor resonance in humans

**DOI:** 10.1038/s41598-023-43227-2

**Published:** 2023-09-23

**Authors:** Giacomo Guidali, Michela Picardi, Maria Franca, Antonio Caronni, Nadia Bolognini

**Affiliations:** 1 Department of Psychology & NeuroMI-Milan Centre for Neuroscience, University of Milano-Bicocca, Piazza dell’Ateneo Nuovo 1, 20126 Milan, Italy; 2 Ph.D. Program in Neuroscience, School of Medicine and Surgery, University of Milano-Bicocca, Monza, Italy; 3 Department of Neurorehabilitation Sciences, Casa di cura Igea, Milan, Italy; 4 Department of Neurorehabilitation Sciences, IRCCS Istituto Auxologico Italiano, Ospedale San Luca, Milan, Italy; 5 Department of Biomedical Sciences for Health, University of Milan, Milan, Italy; 6 Laboratory of Neuropsychology, Department of Neurorehabilitation Sciences, IRCCS Istituto Auxologico Italiano, Milan, Italy

**Keywords:** Cognitive neuroscience, Social neuroscience

## Abstract

In humans, motor resonance effects can be tracked by measuring the enhancement of corticospinal excitability by action observation. Uncovering factors driving motor resonance is crucial for optimizing action observation paradigms in experimental and clinical settings. In the present study, we deepen motor resonance properties for grasping movements. Thirty-five healthy subjects underwent an action observation task presenting right-hand grasping movements differing from their action goal. Single-pulse transcranial magnetic stimulation was applied over the left primary motor cortex at 100, 200, or 300 ms from the onset of the visual stimulus depicting the action. Motor-evoked potentials were recorded from four muscles of the right hand and forearm. Results show a muscle-specific motor resonance effect at 200 ms after movement but selectively for observing a socially relevant grasp towards another human being. This effect correlates with observers’ emotional empathy scores, and it was followed by inhibition of motor resonance at 300 ms post-stimulus onset. No motor resonance facilitation emerged while observing intransitive hand movement or object grasping. This evidence highlights the social side of motor resonance and its dependency on temporal factors.

## Introduction

Assessing corticospinal excitability (CSE) with transcranial magnetic stimulation (TMS) during action observation is considered a classic proxy of activation of the *Action observation network* (AON). In fact, the recruitment of a visuo-motor mirror network is reflected by an enhancement effect of the observer’s corticospinal excitability by action observation, the so-called motor resonance phenomenon^1,2^.

The observation of simple intransitive movements (i.e., made with a single muscle and not goal-directed, like the abduction of a finger) may give rise to motor resonance following mototopic and somatotopic rules, with a maximum activation of motor cortices (namely, the optimal time window to detect the effect) starting from 200 ms from the movement onset^2–5^. Studies using visual stimuli of more complex movements or actions (i.e., made with more than one muscle and goal-directed, like grasping a mug) report controversial findings. Several experimental manipulations could influence motor resonance at a CSE level, from the type of the observed action (e.g., transitive *vs.* intransitive, involved body part, viewing perspective) to the TMS timing, but also participant’s expectations about movement’s outcomes, making it difficult to compare results and paradigms across different studies^6–14^. Hence, optimal parameters for inducing motor resonance in humans are still controversial due to all the factors that could influence its recording^15^. Nevertheless, this investigation is crucial, on the one side, for clarifying the behavioral significance of motor resonance and, on the other, for the successful use of time-locked TMS-based action observation paradigms to study or modulate AON properties exploiting motor resonance as an operative model.

**Figure 1:**
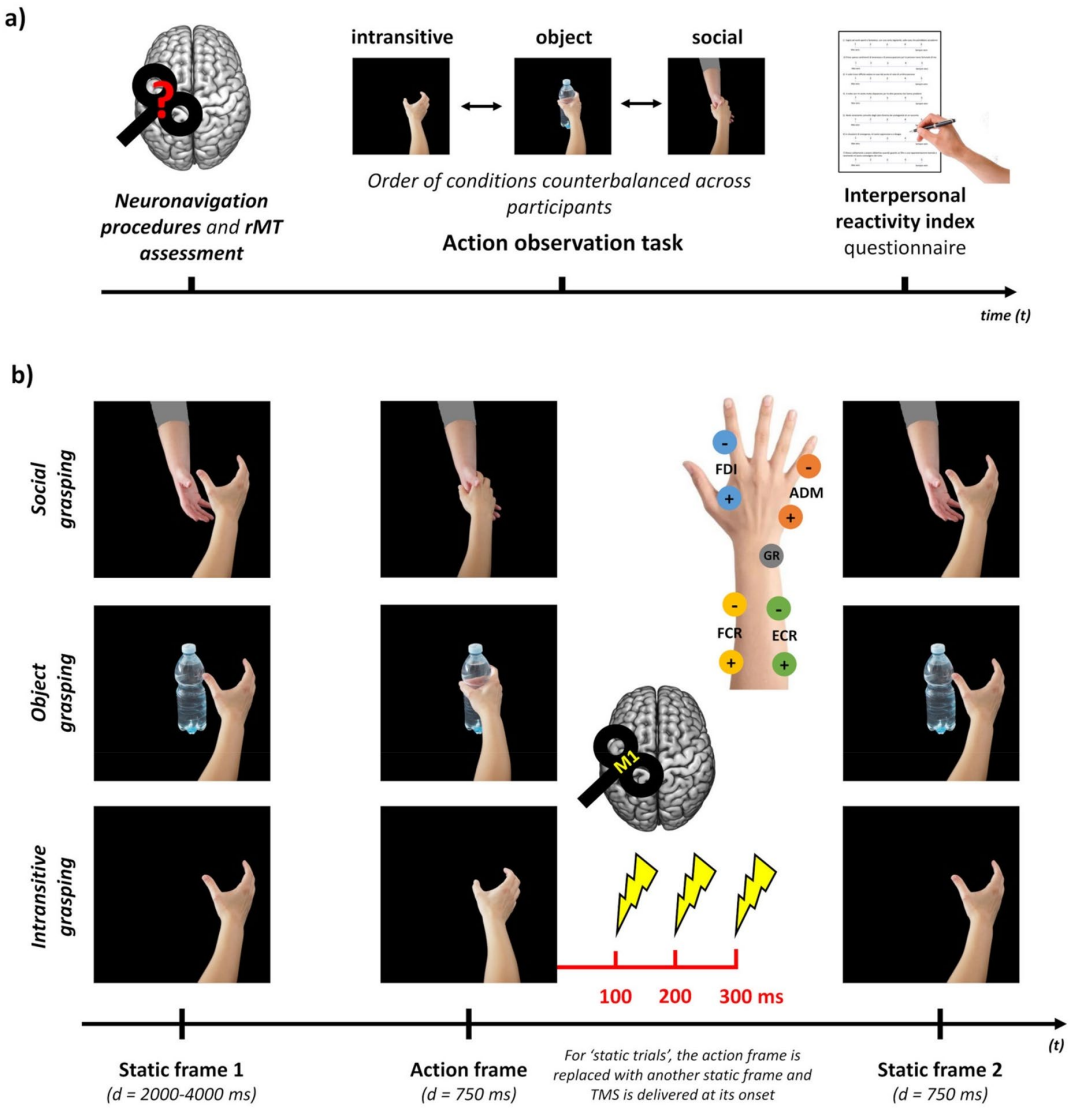
(**a)** *Experimental procedure.* At first, participants underwent neuronavigation procedures and the assessment of the resting motor threshold (rMT). Then, the different blocks of the action observation task were administered. The order of the three experimental conditions (i.e., ‘intransitive grasping’, ‘object grasping’, and ‘social grasping’) was randomized across participants. Finally, the Interpersonal Reactivity Index (IRI) was administered. (**b**) *Action observation task*. The action observation paradigm consisted of a two-frame video-clip showing a right-hand grasping movement whose target was modulated according to the experimental condition. For ‘movement trials’, TMS over left M1 was administered at 120% rMT at 100, 200, or 300 ms from the onset of the action frame. For ‘static trials’—which served as a baseline for detecting motor resonance—the ‘action frame’ was replaced with another ‘static frame’, and TMS was delivered at its onset. In each condition, 136 trials were presented to participants. MEPs were simultaneously recorded from *first dorsal interosseus* (FDI), *abductor digiti minimi* (ADM), *extensor carpi radialis *(ECR), and *flexor* *carpi radialis* (FCR) muscles.

In the present experiment, we deepen motor resonance patterns for different grasping movements in 35 healthy right-handed participants (13 males, mean age ± standard deviation, SD = 22.2 ± 2.1 years; mean education ± SD = 14.7 ± 1.9 years), focusing our investigation on three core elements of motor resonance: (*a*) the target (goal) of the effector’s movement, (*b*) the timing of CSE modulation, and (*c*) the muscle-specificity of the effect. In a two-frame action observation paradigm (i.e., the first frame depicting the static hand, the second frame the action, then the static hand reappeared, thus giving the illusion of apparent motion—Fig. 1), we modulated (*a*) the target stimulus of a right-hand grasping movement observed from an egocentric perspective (i.e., the absence of a target—‘intransitive grasping’ condition, a bottle—‘object grasping’ condition, or a hand—‘social grasping’ condition), and (*b*) the timing of TMS administration over the left primary motor cortex (M1) from the onset of the frame depicting the action (i.e., after 100, 200, or 300 ms) while (*c*) recording motor-evoked potentials (MEPs) from different muscles of the right hand and forearm.

Visual stimuli were selected according to their different level of complexity, i.e., from low to high levels of action representation and motor coding^14^. Namely, in the ‘intransitive grasping’ condition, the grasping act is shown in isolation, without any target or goal. In the ‘object grasping’ condition, the grasping is goal-directed towards an object. Finally, in the ‘social grasping’ condition, the grasping is embedded in a social scenario because participants could interpret it as a social gesture to get in touch with another person. As a baseline to detect motor resonance, we recorded MEPs in each condition while participants observed the frame depicting the static hand. We have selected such a baseline because it allowed us to account for the activation that observing a static biological effector may have per se on CSE or the AON^16^. Our movements were presented in an egocentric perspective and made with the dominant (i.e., right) hand to avoid any spatial remapping onto the observer’s motor systems^15^. Moreover, different studies have shown that AON responses are more significant for actions observed from an egocentric than an allocentric or lateral perspective^17–20^. Finally, the visual stimuli were presented on a black background in all three conditions to prevent the influence of contextual information on CSE. This factor is known to modulate the magnitude of motor resonance effects^21,22^.

The three timings were selected according to previous literature: at 100 ms, some studies investigating the AON already reported non-specific facilitation of CSE or cortical excitability, likely based on the initial, low-level processing of the visual stimulus^23–25^. At 200 ms, different studies converged in finding MEP facilitation during action observation, with muscle-specific patterns of modulation^4–6,26^. Finally, at 300 ms, some studies showed that motor resonance is still detectable, while others reported no effects or reduced ones concerning earlier time points^2,5,27–29^ .

Finally, MEPs were recorded from two muscles of the right hand (i.e., *first dorsal interosseus*—FDI, *abductor digiti minimi*—ADM) and the right forearm (i.e., *extensor carpi radialis*—ECR, and *flexor carpi radialis*—FCR—Fig. 1). Considering the kinematic of our observed stimuli, we suggest that FDI and ECR muscles would be more salient for AON activation than ADM and FCR since their movement is clearly depicted in the visual stimuli^2,14^. Hence, we hypothesized to find greater motor resonance effects for these two muscles.

At the end of the experiment, the Interpersonal reactivity index (IRI), a self-report questionnaire investigating different dimensions of empathy^30^, was administered to participants. Previous studies showed that IRI scores correlated with measures of AON activation^31–35^; hence we were looking for similar patterns also in our motor resonance data.

Motor resonance was quantified as the ratio between MEP peak-to-peak amplitude recorded during the observation of the movement and the one recorded during the observation of the static hand (i.e., our baseline). A series of within-subjects repeated-measures Analysis of Variance (rmANOVA) with factors ‘Condition’ (intransitive grasping, object grasping, social grasping), ‘TMS timing’ (100, 200, 300 ms), and ‘Muscle’ (FDI/ECR, ADM/FCR) were conducted separated for the hand and forearm muscles (see “Methods” for detailed information on the experimental paradigm and analysis).

For completeness, the raw average MEP amplitude in every condition and trial category is reported in Table 1; we also run a control analysis on raw MEP data, including the static conditions along with the movement conditions for direct comparison; this additional analysis is reported in the Supplementary Materials.

**Table 1:** MEP raw data values (mean ± SE, in µV) of the four target muscles in the different experimental conditions and trial categories.

Condition	Trial category	Right-hand muscles MEPs (µV)		Right-forearm muscles MEPs (µV)	
		FDI	ADM	ECR	FCR
Intransitive grasping	Static hand	1959 ± 278	1259 ± 204	893 ± 72	443 ± 52
	100 ms	1936 ± 268	1225 ± 208	898 ± 70	442 ± 49
	200 ms	1972 ± 275	1233 ± 198	908 ± 72	451 ± 54
	300 ms	1907 ± 285	1253 ± 214	888 ± 72	444 ± 54
Object grasping	Static hand	1846 ± 258	1206 ± 190	901 ± 71	448 ± 50
	100 ms	1805 ± 250	1189 ± 183	892 ± 69	442 ± 48
	200 ms	1875 ± 255	1213 ± 186	904 ± 73	443 ± 49
	300 ms	1826 ± 278	1195 ± 193	860 ± 67	432 ± 52
Social grasping	Static hand	1827 ± 223	1122 ± 149	855 ± 59	465 ± 47
	100 ms	1826 ± 238	1119 ± 154	861 ± 58	462 ± 51
	200 ms	2016 ± 244	1143 ± 156	876 ± 65	471 ± 50
	300 ms	1825 ± 250	1115 ± 159	832 ± 58	455 ± 52

## Results

### Motor resonance for hand muscles (FDI/ADM)

The rmANOVA conducted for FDI and ADM muscles showed a significant ‘Condition’ X ‘TMS timing’ X ‘Muscle’ interaction (*F*_4,136_ = 4.19, *p* = 0.003, *η*_*p*_^*2*^ = 0.11), as well as the main effect of ‘TMS timing’ (*F*_2,68_ = 9.79, *p* < 0.001, *η*_*p*_^*2*^ = 0.22) and double interaction ‘TMS timing’ X ‘Muscle’ (*F*_2,68_ = 4.84, *p* = 0.011, *η*_*p*_^*2*^ = 0.13). No other significant effect was found (all *Fs* < 2.69, all *ps* > 0.093). The triple interaction was deepened by conducting a separate rmANOVA for each experimental condition.

**Figure 2:**
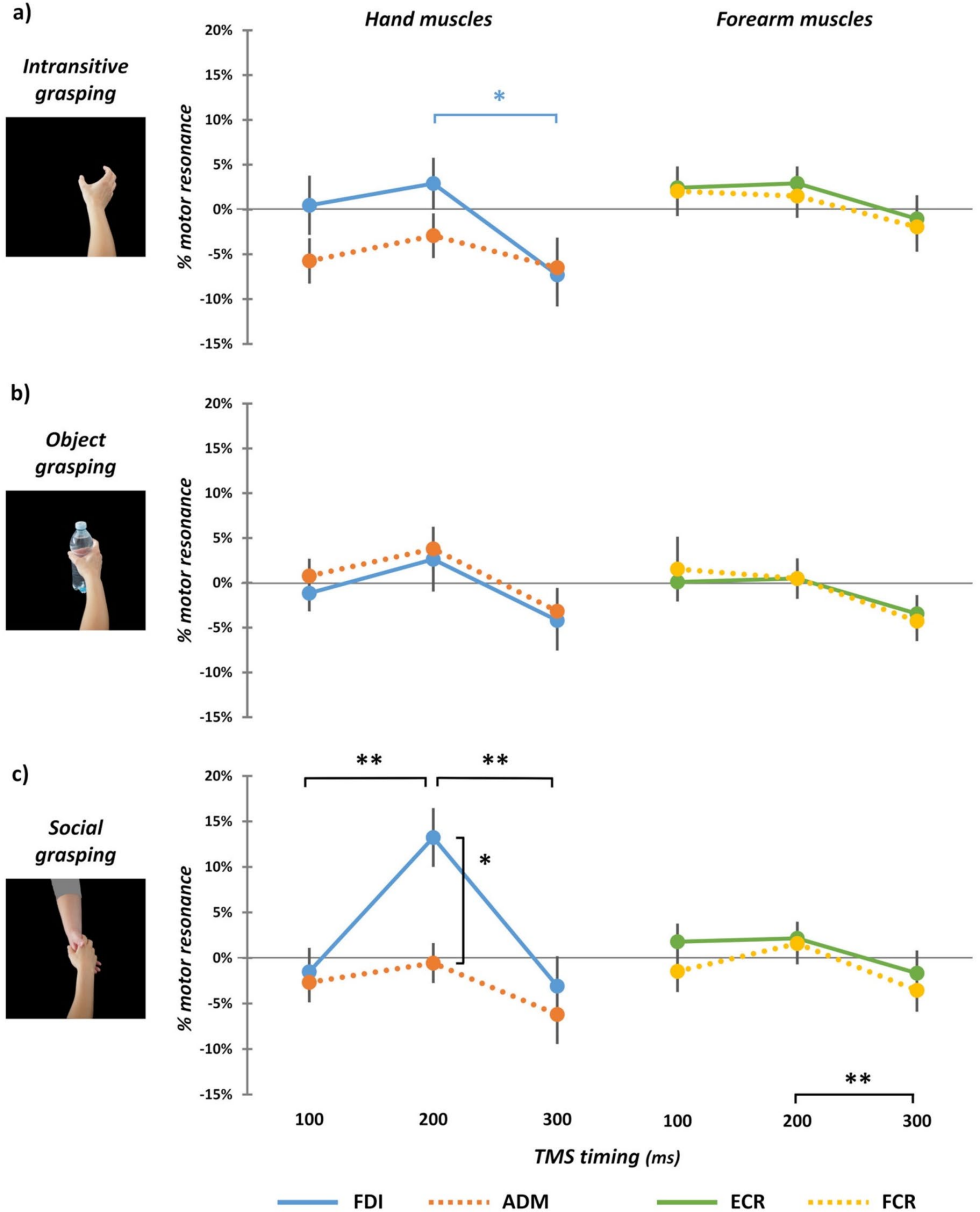
Motor resonance effects (i.e., MEP amplitude in ‘movement trials’ divided for MEP amplitude in ‘static hand trials’) in the three experimental conditions (**a**—‘intransitive grasping’; **b**—‘object grasping’; **c**—‘social grasping’) and for the four muscles (left panels: FDI—straight blue lines, ADM—dotted brown lines; right panels: ECR—straight green lines, FCR—dotted yellow lines) at the three timepoints of TMS administration (100, 200, 300 ms). *p < 0.05; **p < 0.01. Error bars: SE.

Considering the ‘intransitive grasping’ condition, the analysis showed a significant main effect of factor ‘TMS timing’ (*F*_2,68_ = 3.24, *p* = 0.045, *η*_*p*_^*2*^ = 0.09), as well as a significant ‘TMS timing’ X ‘Muscle’ interaction (*F*_2,68_ = 3.99, *p* = 0.023, *η*_*p*_^*2*^ = 0.11). Bonferroni corrected post-hoc comparisons showed that, only for FDI muscle, motor resonance index is significantly different in trials where TMS was delivered after 200 ms for the onset of the movement (mean ± standard error—SE: 2.89 ± 2.88%) compared to trials in which the timing was 300 ms (− 7.29 ± 3.53%; *t*_35_ = 3.17; *p* = 0.048, *d* = 0.54; Fig. 2a).

Considering the ‘object grasping’ condition, neither the main factors (‘TMS timing’: *F*_2,68_ = 3.04, *p* = 0.054, *η*_*p*_^*2*^ = 0.08; ‘Muscle’:* F*_1,34_ = 0.38, *p* = 0.54, *η*_*p*_^*2*^ = 0.01) nor the interaction (*F*_2,68_ = 0.06, *p* = 0.939, *η*_*p*_^*2*^ < 0.01) reached statistical significance, highlighting the absence of motor resonance effects in this condition (Fig. 2b).

Crucially, considering the ‘social grasping’ condition, both factors (‘TMS timing’: *F*_2,68_ = 8.62, *p* < 0.001, *η*_*p*_^*2*^ = 0.2; ‘Muscle’:* F*_1,34_ = 4.7, *p* = 0.037, *η*_*p*_^*2*^ = 0.12) and their interaction (*F*_2,68_ = 7.47, *p* = 0.001, *η*_*p*_^*2*^ = 0.18) reached statistical significance. Post-hoc showed that the motor resonance index obtained for FDI muscle in trials where TMS was delivered after 200 ms from the movement onset (13.23 ± 3.24%) was significantly higher than in all the other TMS timing, both for FDI (v*s.* 100 ms: − 1.52 ± 2.63%; *t*_35_ = 4.64; *p* < 0.001, *d* = 0.78; vs. 300 ms: − 3.09 ± 3.29%; *t*_35_ = 3.99; *p* = 0.005, *d* = 0.68) and ADM (vs. 100 ms: − 2.68 ± 2.21%; *t*_35_ = 3.96, *p* = 0.005, *d* = 0.67; vs. 200 ms: − 0.56 ± 2.20%; *t*_35_ = 3.51, *p* = 0.019, *d* = 0.59; vs. 300 ms: − 6.2 ± 3.26%; *t*_35_ = 4.07, *p* = 0.004, *d* = 0.69—Fig. 2c). This evidence suggests that the observation of a social grasping enhances MEPs amplitude only when TMS is delivered at 200 ms and, thus, that this is the optimal timing to detect facilitation of CSE.

### Motor resonance for forearm muscles (ECR/FCR)

Results from the rmANOVA conducted on the ‘% motor resonance’ index for ECR and FCR muscles did not show a significant triple interaction ‘Condition’ X ‘TMS timing’ X ‘Muscle’ interaction (*F*_3.06,104.2_ = 0.43, *p* = 0.737, *η*_*p*_^*2*^ = 0.01). Indeed, only a significant main effect of factor ‘TMS timing’ was found (*F*_2,68_ = 5.04, *p* = 0.009, *η*_*p*_^*2*^ = 0.13). In detail, regardless of the muscle or the condition, motor resonance when TMS was delivered after 300 ms from the movement onset (− 2.35 ± 1.38%) was lower than when TMS timing was 200 ms (1.51 ± 0.97%; *t*_35_ = − 2.81, *p* = 0.024, *d* = 0.55; Fig. 2a–c). No other significant effect was found (all *Fs* < 0.38, all *ps* > 0.687), suggesting that for forearm muscles, no motor resonance was detectable during the observation of our grasping movements.

### Participants’ emphatic scores and motor resonance patterns

**Figure 3:**
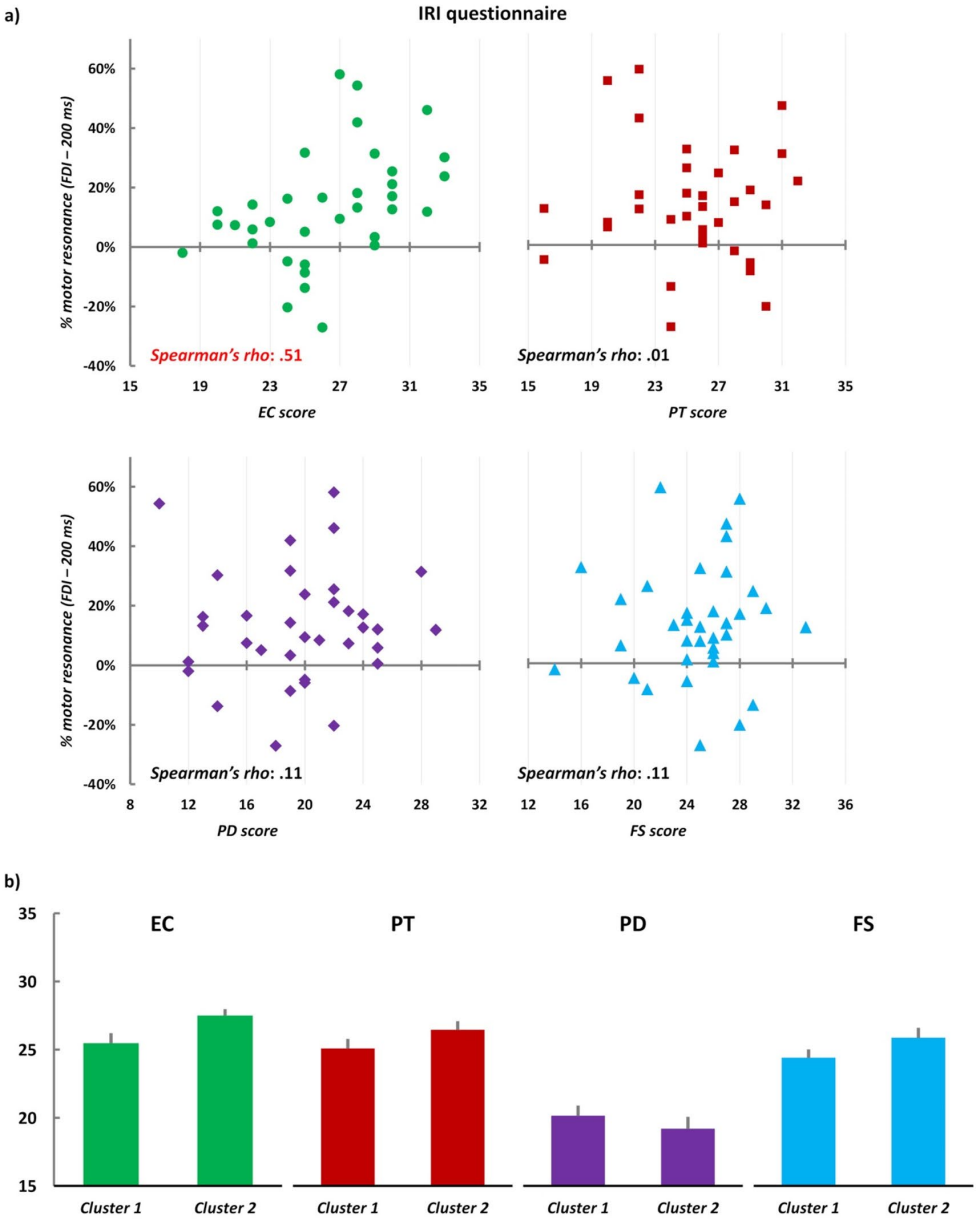
(**a**) Scatterplots between participants’ scores in the IRI’s four subscales (x-axis) and motor resonance effect for FDI muscle at 200 ms timing (y-axis). A significant correlation (*Spearman’s rho*) is found only for the EC scale. (**b**) IRI scores in the two clusters of our sample found running hierarchical clustering. No statistically significant differences were found between clusters. Error bars: SE.

As shown in Fig. 2c, a significant motor resonance effect was found only in the ‘social grasping’ condition for MEPs recorded from FDI and when TMS was delivered at 200 ms from the onset of the action frame. Hence, we correlated the magnitude of this effect with participants’ scores in the four subscales of the IRI (i.e., *emphatic concern*—EC, *perspective taking*—PT, *fantasy*—FS, and *personal distress*—PD). The only significant correlation was found for the EC subscale: the higher the participant’s score in this subscale, the higher the motor resonance effect (*Spearman’s rho* = 0.51, *p* = 0.002). None of the other correlations reached the significance level (PT: *Spearman’s rho* = 0.01, *p* = 0.952; FS: *Spearman’s rho* = 0.11, *p* = 0.513; PD: *Spearman’s rho* = 0.11, *p* = 0.549; Fig. 3a). However, the significant correlation between EC scores and motor resonance facilitation for social stimuli could be due to a subgroup of participants with higher levels of empathy and, thus, cannot be generalized to the whole population. To explore this possibility, we have run a hierarchical cluster analysis^36^ on participants’ motor resonance effects at 200 ms timing. This analysis revealed two clusters of participants (see “Methods”), but these clusters did not differ either for the four IRI subscales scores (EC: *F*_1,34_ = 2.38, *p* = 0.132, *η*_*p*_^*2*^ = 0.07; PT: *F*_1,34_ = 0.95, *p* = 0.336, *η*_*p*_^*2*^ = 0.03; FS.: *F*_1,34_ = 1.13, *p* = 0.296, *η*_*p*_^*2*^ = 0.03; PD: *F*_1,34_ = 0.33, *p* = 0.570, *η*_*p*_^*2*^ = 0.01—Fig. 3b) or for the IRI total score (*F*_1,34_ = 1.23, *p* = 0.275, *η*_*p*_^*2*^ = 0.04); this finding allows to exclude that the empathic scores of a subset of participants could be responsible of the modulation of motor resonance at 200 ms.

## Discussion

Our results show that a clear-cut CSE facilitation resembling motor resonance is detectable only for social grasping actions (i.e., when the grasping movement is directed towards another hand). It occurs after 200 ms from action-observation onset and only for the FDI muscle. Furthermore, at 300 ms, motor resonance is significantly lower than at 200 ms, and this inhibition-like effect is present regardless of the type of viewed action. Specifically, the inhibition of CSE is hand-muscle-specific (being present for FDI but not for ADM muscle) only in the ‘intransitive’ and ‘social’ grasping conditions. Conversely, for both the forearm muscles (ECR and FCR), this inhibitory pattern at 300 ms is found in every condition. These temporal effects argue the model proposed by Naish and colleagues (2014) in their review on timing-dependent modulation patterns of CSE during action observation^2^. In fact, at 100 ms, we do not find facilitation of MEPs in any of our conditions. In contrast, at 300 ms, motor resonance is significantly lower than at 200 ms, suggesting that, at this later timing, and at least for the sight of hand movement with or without a goal, CSE is inhibited regardless of the muscle involved in the observed action.

The critical result is that muscle-specific facilitation of MEPs—reflecting motor resonance—could be observed only at 200 ms and for the ‘social grasping’ condition, i.e., when the movement targets another hand. In line with previous literature^2^, this modulation is specific for FDI, i.e., the principal muscle involved in the grasping movement depicted. As said in the “Introduction”, this kind of visual movement stimulus activates high-level aspects of action representation, like those with a social-related value^14^. This evidence is corroborated by different functional magnetic resonance imaging (fMRI) studies, which show that observing target-directed actions, as well as actions with a social valence, activated to a greater extent than non-object related ones fronto-parietal regions of the AON^37,38^. This greater activation may enhance the sensorimotor system and then CSE, favoring the detection of motor resonance for this kind of visual stimuli^39–41^. For instance, Streltsova and coworkers (2010), using electroencephalography (EEG), showed that the observation of a social grasping (i.e., a hand grasping a ball from another’s hand) triggered an event-related alpha desynchronization over sensorimotor electrodes—an EEG marker of motor resonance—with a more dynamic time course than the one triggered by the observation of a simple grasping (i.e., and hand grasping a ball). This evidence suggests the activation of different motor resonance mechanisms according to the social relevance of the observed hand behavior^41^. Indeed, it has been proposed that one of the hallmark functions of the *mirror neuron system* is to serve social perception and interactions thanks to the existence of *other-selective* mirror neurons implicated in the control of one’s behavior and its coordination with others during social interactions (the so-called ‘social affordance’ framework^42–44^). Our results support this view, suggesting that actions with a social significance/goal, as happens in the social grasping condition, are crucial to obtaining a stable readout of motor resonance facilitation (and, thus, of AON recruitment).

Notably, the motor resonance facilitation found in the social grasping condition cannot be attributable to a higher salience of such a visual stimulus as compared to intransitive and transitive (object-directed) actions^45^. Indeed, the additional analysis of the raw MEPs (see Supplementary Materials) showed the absence of significant modulation of MEP amplitudes during the observation of every type of static stimulus (Supplementary Fig. 1), in turn confirming that the CSE increase was due to the sight of a social movement. The mere observation of static hands with a social significance (see the static frame illustrated in Fig. 1), although of potentially higher salience and interest for the observer, has no effect on CSE.

The social significance of motor resonance is also supported by the positive correlation between the magnitude of the motor resonance effect and participants’ scores in the EC subscale of the IRI, a scale measuring emotional empathy and the tendency to feel the emotions of others^30^. The absence of selective motor resonance effects for subgroups of individuals with different (high or low) empathic traits, as shown by the cluster analysis, suggests that the social modulation of motor resonance at 200 ms is directly ascribable to the type of observed action, rather than to individual factors. Previous studies have shown that different subscales of this questionnaire correlated with measures of mirror neuron system activation^32–34^. For instance, in an fMRI study, Kaplan and Iacoboni report a significant correlation between participants’ EC scores and the activity in the inferior frontal gyrus, a key area of the AON, suggesting that the AON subserves to the analysis of other person’s intentions to access their emotional states^33^. Hence, considering our results, participants with high emotional empathy have a more activated AON at the sight of the social grasping visual stimulus, and this is reflected, at a neurophysiological level, in a more robust modulation of CSE and motor resonance.

The other interesting result is that motor resonance is significantly lower at 300 ms than at 200 ms, especially for the intransitive and social grasping actions, highlighting a CSE inhibition during action observation that follows the motor resonance effect (see Table 1 and Supplementary Materials). Studies in the monkeys showed the presence of mirror neurons in M1 and the premotor cortex (F5 area) that suppressed their activity during grasping observation, leading to inhibitory effects in corticospinal circuitry^46,47^. In humans, similar inhibitory patterns were found during action observation using CSE-based motor resonance indexes^29,48,49^. Overall, this evidence suggests ﻿a suppression of motor activity in the muscles involved in the viewed action to prevent the overt movement in the observer. Hence, observing a movement initially increases motor cortex excitability in the observer through AON recruitment, giving rise to motor resonance facilitation. However, motor resonance is inhibited whenever the observer refrains from imitating the observed movement (as in our action observation task), and in turn, motor cortex excitability decreases^2^. Our results confirm the existence of this ‘dual’ mirroring process, also showing that the suppression effect is target- and muscle-specific as the motor resonance one, at least for hand muscles. Noteworthy, such specificity could also explain why previous studies did not always find inhibitory effects during action observation.

The movement depicted in our action stimuli allows us to disentangle why, for the forearm muscles, suppression effects at 300 ms were found regardless of the grasping condition or the recorded muscle (see Fig. 2). Our grasping stimuli also include a reaching component with the forearm. Thus, the AON suppression effect to prevent imitation may be easier to detect for muscles that initiated the observed movement (i.e., ECR and FCR, that are primarily involved in the initial reaching stage of the observed grasping action) rather than for finger muscles that are more relevant for the later grasping stage of the action. Future studies are needed to better shed light on this hypothesis.

With respect to the object grasping and intransitive conditions, unlike previous literature^2^, we did not detect any CSE facilitation. However, at variance with those studies reporting motor resonance facilitation even during the observation of intransitive or object-related grasping movements^1,11,27,50,51^, here we used an action observation task comprising only one picture for the action stimulus, rather than multi-frame video-clips (Fig. 1 and “Methods”). In particular, similar to other action observation tasks presenting elementary intransitive movements like a finger abduction^5,52,53^, our visual action stimuli consisted in the rapid succession of one ‘static’ frame followed by one ‘action’ frame, which gives rise to a movement illusion. We made such a choice to better control the timing of the TMS-driven activation of M1 by action observation. However, this strategy could have influenced the chance to track motor resonance effects according to the dynamic evolution of the viewed action (see, e.g.,^50,54^—the first showing that as the hand gets closer to the object, the CSE increase), consequently explaining the lack of faciliatory effect during intransitive and transitive action observation.

For instance, Urgesi and colleagues (2010) found that CSE facilitation during the observation of grasping snapshots is maximal when the movement is ongoing—or at its start—rather than at its end, suggesting the potential role of anticipatory simulation of the future action phase^54^. Such an anticipatory component of motor resonance cannot be monitored with our paradigm since the CSE modulation was explored in every condition only after the ‘action’ frame—which depicts the grasping movement completed.

Moreover, the static hand could be interpreted as ‘*a hand ready to move/grasp’* (i.e., an action at its starting phase—Fig. 1), but since participants, after a few trials, become familiar with a specific movement (i.e., grasping), the facilitation of CSE could not occur—or it could be reduced—because the AON is already pre-activated at the mere sight of the effector (i.e., static hand). In this regard, it has to be noted that previous works showed that the implied motion present in the static hand—which we exploited as a baseline to calculate our index of motor resonance—could lead to the extinguish of CSE facilitation during the observation of movements, likely through predictive mechanisms^10,54,55^. We can hypothesize that for action with low-level motor coding (i.e., simple action kinematics or actions towards an object), the implied motion during the observation of the static hand, as well as anticipatory simulation mechanisms, plays a crucial role in influencing motor resonance patterns. Conversely, for stimuli embedding high-level aspects of action representation—as our ‘social grasping’ condition, the pattern follows the expected one (i.e., facilitation of MEPs) even when the static hand is used as a baseline or anticipatory mechanisms could be potentially involved. Nevertheless, this remains speculation, and future studies are needed to disentangle better the pattern of results found for intransitive and object grasping conditions.

With respect to the absence of CSE facilitation for forearm muscles, as well as the ADM muscle, some methodological aspects should be considered. Firstly, as described in the “Methods” section, during our action observation paradigm, participants had to detect the presence of (sporadic) colored dots, which could appear in a fixed position on the back of the observed grasping hand. This task was introduced to ensure participants didn’t close their eyes or fall asleep. However, in this way, participants’ attention was directed on the hand rather than on the forearm, which could have favored motor resonance modulations in the hand (FDI, ADM) muscles rather than in the forearm (ECR, FCR) ones. In fact, it has been shown that the observer’s locus of attention influences the magnitude of CSE modulation and AON activation^56–59^.

Secondly, since during the actual execution of whole-hand grasping movements ADM is also involved^60^, motor resonance modulation patterns should also have been found for this hand muscle, at least in the social condition. Nevertheless, the absence of motor resonance modulation for ADM could be attributable to the visual characteristics of our stimuli. Our grasping movement is presented in an egocentric perspective, and the movement of the hypothenar hand region (and thus of the little finger) is only suggested by the presumed kinematic of the grasping. Still, our visual stimuli do not clearly depict it (see Fig. 1). This may have then maximized motor resonance for FDI (whose movement is in the foreground) rather than ADM, hence detecting a motor resonance effect confined to the FDI muscle (for similar muscle-specific results, see e.g.,^7,61,62^). In a broader perspective, this evidence highlights once more the muscle- and visual-specificity of motor resonance and, thus, the critical importance of taking into account visual stimuli characteristics exploited in the action observation task to replicate previous findings and patterns.

In conclusion, the present experiment confirmed that motor resonance is a dynamic and flexible phenomenon that does not reflect the inner replica of the observed movement, at least when complex, transitive movements are presented to participants^14^. A clear-cut enhancement of CSE during action observation is found only for the grasping movement conveying social information (i.e., grasping another hand), and this modulation has a time course that reflects the one found in previous literature for intransitive, muscle-specific movements. This facilitation is then followed by motor resonance inhibition at 300 ms, which could be related to movement suppression during action observation^2^. Our results are also relevant from a methodological perspective: different task-related factors may influence motor resonance effects, impacting the effectiveness of the exploited action observation paradigm.

## Methods

### Participants

Forty healthy volunteers were enrolled in the study; one of them was excluded because he/she was left-handed, two due to recording problems during the experimental session, and two presented outlier values (i.e., >  ± 2.5 SD—from the mean of the condition) in at least one of our dependent variables. Thus, the final analyzed sample was 35 participants (13 males, mean age ± SD = 22.2 ± 2.1 years; mean education ± SD = 14.7 ± 1.9 years). The sample size was determined with the software G*Power 3.1^63^, using an a-priori within-subjects rmANOVA and setting an estimated medium effect size of η_p_^2^ = 0.06 with alpha error level: *p* = 0.05 and statistical power of 0.9. With these parameters, the recommended sample size to achieve enough statistical power was 33 participants. According to the Edinburgh handedness inventory^64^, participants were all right-handed (mean score ± SD = 80.7 ± 16.4%), and none had contraindications to TMS^65^. Before taking part in the study, participants gave their written informed consent. The experiment was performed following the ethical standards of the Declaration of Helsinki and the Ethical Committee of the University of Milano-Bicocca approved it. Participants received 0.3 European university credits (ECTS) for participating.

### Action observation paradigm

CSE, and hence the motor resonance effect, was measured by recording MEPs induced by the stimulation of the left M1. MEPs were recorded from two muscles of the right hand, i.e., FDI and ADM, and two muscles of the right forearm, i.e., ECR and FCR. MEPs were collected while participants observed video clips showing static or moving hands depicting a grasping movement made with the right hand in an egocentric perspective. The action observation paradigm exploited is a modified version of a task already exploited by our research group in previous studies on motor resonance with intransitive finger movements^52,53^.

Participants were seated in a chair in front of a PC screen, approximately 57 cm from their faces, with their hands relaxed on a table and out of view. The task consisted of three blocks, presented in a randomized order, in which the target of the grasping movement varied (i.e., a bottle—‘object grasping’ condition, a hand—‘social grasping’ condition, or the movement without any target—‘intransitive grasping’ condition). Every trial began with a static hand presented in an egocentric perspective and on the right side of the screen for a variable duration from 2 to 4 s. In the ‘object’ and ‘social grasping’ conditions, the target was also presented in this frame centrally on the screen and in the background concerning the static hand. Then, a second frame (i.e., action frame) was presented with a fixed duration of 750 ms. In ‘movement trials’, the frame showed the grasping movement of the right hand. Concerning the task’s condition, this movement could not be directed to any target (‘intransitive grasping’ condition), directed to a bottle (‘object grasping’ condition), or a human’s hand (‘social grasping’ condition). Regardless of the experimental condition, TMS could be delivered at three different times according to the onset of the action frame: (*a*) after 100 ms, (*b*) after 200 ms, or (*c*) after 300 ms. In ‘static hand trials’, the second frame of the task was still showed, but the hand remained static (i.e., the same visual stimulus as the first frame). Here, TMS was delivered at its onset. In all trials, TMS intensity was set at 120% of the participant’s rMT—see next paragraph. Before data collection, we checked the timing of TMS pulses from the action frame using a photodiode. Finally, the frame depicting a static hand appeared again for 750 ms, ending the trial (Fig. 1b).

For each condition, 136 trials were presented to the participants. Thirty-four were ‘static hand trials’, and 102 were ‘movement trials’; of the latter ones, 34 had TMS delivered after 100 ms from the ‘action frame’, 34 after 200 ms, and 34 after 300 ms. Trials presentation of each condition was separated into two further sub-blocks of 68 trials (comprising 17 trials for each category mentioned above) lasting about 5 min with a brief pause in the middle. To ensure that participants kept attention to the visual stimuli, in each condition of the action observation task, 16 trials out of 136 (i.e., four trials for each category) presented a small red dot (diameter: 20 pixels) that appeared on the back of the right hand during the second frame of the task. Participants had to press with their left hand one of the PC mouse keys every time the dot appeared. On average, participants’ accuracy in this attentive task was 95.5% (SD =  ± 3.2%). MEPs recorded during these attentional trials were not analyzed.

Timing of the stimuli and trials randomization were presented under computer control using the software E-Prime 2.0 (Psychology Software Tool, Inc.).

### TMS and electromyography (EMG) recording

Biphasic TMS pulses were delivered with a figure-of-eight coil (diameter = 70 mm) connected to a Magstim Rapid 2 stimulator (Magstim, Whitland, UK). The motor hotspot of the right-hand FDI muscle was found by moving the coil in 0.5 cm steps around the presumed motor hand area of the left hemisphere by using a slightly supra-threshold stimulus and recording MEPs. For all four muscles, active electrodes (15 × 20 mm Ag–AgCl pre-gelled surface electrodes, Friendship Medical, Xi'an, China) were placed over the muscle bellies. For FDI and ADM, reference electrodes were placed over the metacarpophalangeal joint of the index finger and the little finger, respectively; considering ECR and FCR, they were placed on the muscle–tendon at a distance of about 5 cm from the active electrode. The ground electrode was placed over the right head of the ulna. Before data acquisition, experimenters made a visual inspection to check that background noise from the four channels was smaller than 50 µV. MEP signal was acquired applying a 50 Hz notch filter and a sample of 5000 Hz, amplified, band-pass filtered (10–1000 Hz), and stored for offline analysis. Data were collected from 100 ms before to 200 ms after the TMS pulse (time window: 300 ms). MEPs were recorded using Signal software (version 3.13) and a Digitimer D360 amplifier with a CED micro1401 A/D converter (Cambridge Electronic Devices, Cambridge, UK).

The individual rMT was calculated by employing the parameter estimation by sequential testing (PEST) procedure, which is a maximum-likelihood threshold-hunting procedure optimized for rMT detection (Awiszus, 2003; Dissanayaka et al., 2018). On average, participants presented an rMT of 60.9% (SD =  ± 9.8%). Given that we used a single muscle (i.e., FDI) as reference for the motor hotspot (and, hence, to determine rMT), before starting the action observation task, we ensured that the participants presented stable MEPs of at least 200 µV in all the four target muscles at the stimulation intensity employed. All the participants respected this criterion.

For M1 stimulation, the coil was placed tangentially to the scalp with the handle held backward and laterally at a 45° angle to the sagittal plane, inducing currents in the brain with an anterior-to-posterior (first phase)/posterior-to-anterior (second phase) direction. The stable TMS coil placement and position were monitored during the experimental sessions using the SofTaxic Optic 3.4 neuronavigation software (EMS, Bologna, Italy).

### Interpersonal reactivity index questionnaire (IRI)

The IRI is a self-report questionnaire that comprises four subscales of 7 items, each measuring a separate aspect of empathy (Ref.^30^, for italian validation, see Ref.^66^). In detail, the four subscales are (*a*) *empathic concern* (EC, i.e., ‘other-oriented’ feelings of sympathy and concern for others), (*b*) *personal distress* (PD, i.e., ‘self-oriented’ feelings of personal anxiety and unease in tense interpersonal setting), (*c*) *perspective taking* (PT, i.e., tendency to adopt the psychological point of view of others), and (*d*) *fantasy* (FS, i.e., tendency to transpose oneself imaginatively into the feelings of fictitious characters). EC and PD subscales measure affective empathy, whereas PT and FS measure cognitive empathy^66^. Higher scores indicate a higher level of empathy. Participants report the extent to which each of the 28 statements describes oneself on a 5-point Likert scale ranging from 0 (‘Does not describe me well’) to 4 (‘Describes me very well’)^30^. IRI compilation takes about 5–10 min.

### Experimental procedure

The experiment started with administering the informant consensus, the Edinburgh handedness inventory, and the TMS checklist questionnaire. After these, neuronavigation procedures were carried out, and the individual rMT was assessed. Then, the action observation task was administered. The order of the blocks with the three conditions of the task (i.e., ‘intransitive grasping’, ‘object grasping’, and ‘social grasping’) was randomized across participants following a Latin square design (i.e., ABC, BCA, or CAB). Finally, the IRI was administered (Fig. 1a). On average, a session lasted 1 h and 30 min.

### Statistical analysis

MEPs were analyzed offline using Signal software (version 3.13, Cambridge Electronic Devices, Cambridge, UK) exploiting the standard pipeline currently used in our laboratory^52,53^. Trials with artifacts (i.e., muscular or background noise) deviating from 200 µV in the 100 ms before the TMS pulse were excluded from the analysis. For each muscle and in each trial, MEPs peak-to-peak amplitude was calculated in the time window between 5 and 60 ms from the TMS pulse. For each condition and each muscle, trials in which MEPs amplitude was ± 2.5 SD from the mean of each trial type (i.e., ‘static hand trials’, ‘movement trials’ with TMS after 100 ms, ‘movement trials’ with TMS after 200 ms, ‘movement trials’ with TMS after 300 ms) were considered outliers and thus excluded from the analysis. Motor resonance effects were computed as the ratio in MEP amplitude between movement and static conditions subtracted by one:

$$\% \; motor \; resonance=\frac{MEP \; amplitude \; in \; movement \; trials}{MEP \; amplitude \; in \; static \; trials} -1$$

In all three blocks and for the four muscles, the mean MEP amplitude in the three different conditions of movement trials (i.e., with TMS after 100, 200, or 300 ms) was divided for MEP amplitude in static hand trials. The value ‘1’ was subtracted from the ratio to express the percentage concerning the static condition. Hence, positive percentages indicate CSE facilitation by action observation, while negative ones indicate CSE inhibition by action observation. All subsequent analyses were conducted using such an index.

Data analyses were performed to detect motor resonance modulation through a series of within-subjects rmANOVA split for the muscles of the hand (FDI, ADM) and the muscles of the forearm (ECR, FCR). These rmANOVAs had the factors ‘Condition’ (intransitive grasping, object grasping, social grasping), ‘TMS timing’ (100 ms, 200 ms, 300 ms), and ‘Muscle’ (FDI/ECR, ADM/FCR). If the triple interaction would be significant, we explored the modulation patterns in each condition through a ‘TMS timing’ X ‘Muscle’ rmANOVA with the same levels of the factors mentioned earlier. Then, if a statistically significant motor resonance effect would be found in one of these analyses, we correlated the effect (i.e., % motor resonance) with the four subscales of the IRI using the *Spearman* correlation coefficient, looking for possible relations between MEPs facilitation and participants’ emphatic scores.

Finally, considering the results of the previous analysis (see “Results”), to better disambiguate whether motor resonance effects at 200 ms could be driven by a subgroup of high empathic participants—and hence they cannot be generalized to our whole sample, we also run a hierarchical cluster analysis on standardized data (distance measure: Euclidean, clustering method: Ward.D2) using *snowCluster* module^67^ on the software Jamovi (version 2.3.21, www.jamovi.org) and considering each participant as a point in space described by the following dimensions: % motor resonance at 200 ms during the three conditions and the four muscles, and the IRI scores. Dendrogram analysis suggested the presence of two clusters of participants with a numerosity of 14 and 21, respectively. Then, we run an additional one-way ANOVA with between-subjects factor ‘Cluster’ (1, 2) to explore whether IRI scores were statistically different in the two clusters found.

All statistical analyses were performed using the software Jamovi^68^. Statistical significance was set at *p* < 0.05. Normality of the distributions was confirmed for all our variables, checking it with the Shapiro–Wilk test and Q-Q plots assessment. For rmANOVAs, data sphericity was tested by applying Mauchly’s test in every dataset. When data sphericity was not confirmed, the Greenhouse–Geisser correction was applied. Significant main effects and interactions were further explored by applying the Bonferroni correction for multiple comparisons. Partial eta-squared (*η*_*p*_^*2*^) and Cohen’s *d* were calculated in every rmANOVA and t-test, respectively, and reported as effect size values. If not otherwise specified, in the “Results” section, mean ± SE is reported for each variable.

### Supplementary Information

Supplementary Information.

## Data Availability

Datasets, analysis, tasks, and stimuli used in the experiment are publicly archived at the Open Science Framework (OSF): https://osf.io/mksg5/. Further information will be available from corresponding authors upon reasonable request.
