# Author Correction: The social relevance and the temporal constraints of motor resonance in humans

**DOI:** 10.1038/s41598-024-61228-7

**Published:** 2024-05-06

**Authors:** Giacomo Guidali, Michela Picardi, Maria Franca, Antonio Caronni, Nadia Bolognini

**Affiliations:** 1 Department of Psychology and NeuroMI‑Milan Centre for Neuroscience, University of Milano-Bicocca, Piazza Dell’Ateneo Nuovo 1, 20126 Milan, Italy; 2 Ph.D. Program in Neuroscience, School of Medicine and Surgery, University of Milano-Bicocca, Monza, Italy; 3 Department of Neurorehabilitation Sciences, Casa Di Cura Igea, Milan, Italy; 4 Department of Neurorehabilitation Sciences, IRCCS Istituto Auxologico Italiano, Ospedale San Luca, Milan, Italy; 5 Department of Biomedical Sciences for Health, University of Milan, Milan, Italy; 6 Laboratory of Neuropsychology, Department of Neurorehabilitation Sciences, IRCCS Istituto Auxologico Italiano, Milan, Italy

Correction to: *Scientific Reports*, 10.1038/s41598-023-43227-2, published online 23 September 2023.

The original version of this Article contained an error in the Funding section.

“Research funded by the Italian Ministry of Health; G.G. has been supported by the grant ‘Fondo di Ateneo Quota Competitiva’ (022-ATEQC-0005) of the University of Milano-Bicocca.”

now reads:

“The publication fee has been supported by Ricerca Corrente from Italian Ministry of Health - G.G. has been supported by the grant ‘Fondo di Ateneo Quota Competitiva’ (022-ATEQC-0005) of the University of Milano-Bicocca to NB.”

The original Article has been corrected.

