# Supplementary material for: The social relevance and the temporal constraints of motor resonance in humans
Source: Sci Rep. 2023 Sep 23;13:15933. doi: 10.1038/s41598-023-43227-2 (PMC10517949; doi:10.1038/s41598-023-43227-2)
Supplement: Supplementary file 1 — Supplementary Information. [file 41598_2023_43227_MOESM1_ESM.pdf]

The social relevance and the temporal constraints of motor resonance in humans

Giacomo Guidali, Michela Picardi, Maria Franca, Antonio Caronni & Nadia Bolognini

SUPPLEMENTARY MATERIALS

Analysis on raw MEPs values

To further investigate the pattern of results found on motor resonance, we also run rmANOVAs on raw MEP amplitudes (see **Table 1** in the main text for their mean values). Considering the not normal distribution of these variables (i.e., Shapiro-Wilk normality test < .05), we have transformed them by applying base-ten logarithm [i.e., $\log_{10}$(MEP amplitude)], in turn making the distributions closer to normality (i.e., Shapiro-Wilk normality test > .05). Data analyses were performed following the same methodology described in the main text, namely through a series of within-subjects rmANOVA split for the muscles of the hand (FDI, ADM) and the muscles of the forearm (ECR, FCR), with the within-subjects factors: 'TMS administration' (now 4-levels, to include the static hand, as well as movement 100 ms, movement 200 ms, movement 300 ms), 'Condition' (intransitive grasping, object grasping, social grasping), and 'Muscle' (FDI/ECR, ADM/FCR). Data sphericity was tested by applying Mauchly's test. When data sphericity was not confirmed ($p < .05$), the Greenhouse-Geisser correction was applied.

The rmANOVA conducted for FDI and ADM muscles showed a significant 'Condition' X 'TMS administration' X 'Muscle' interaction ($F_{4.48,152.24} = 2.9$, $p = .02$, $\eta_p^2 = .08$), as well as the main effect of 'TMS administration' ($F_{1.83,62.25} = 8.66$, $p < .001$, $\eta_p^2 = .2$), 'Muscle' ($F_{1,34} = 38.31$, $p < .001$, $\eta_p^2 = .53$) and double interaction 'TMS administration' X 'Muscle' ($F_{3,102} = 3.67$, $p = .015$, $\eta_p^2 = .1$). Crucially, main effect of factor 'Condition' was not statistically significant ($F_{2,68} = .45$, $p = .638$, $\eta_p^2 = .01$), suggesting that CSE is not overall modulated by the type of visual stimulus of the action observation task. Moreover, planned comparisons (Bonferroni corrected) conducted for 'static hand' trials showed that ($\log_{10}$) MEP amplitudes did not differ among grasping conditions (for FDI: intransitive *vs.* object: $t_{34} = -.07$; $p = .948$; $d = .01$; intransitive *vs.* social: $t_{34} = -.56$; $p = .581$; $d = .09$; object *vs.* social: $t_{34} = -.67$; $p = .51$; $d = .11$; for ADM: intransitive *vs.* object: $t_{34} = -.14$; $p = .89$; $d = .02$; intransitive *vs.* social: $t_{34} = -.14$; $p = .887$; $d = .02$; object *vs.* social: $t_{34} = -.03$; $p = .973$; $d < .01$; **Supplemental Figure 1a**). No other significant effect was found (all *Fs* < 1.9, all *ps* > .157).

The rmANOVA for ECR and FCR muscles did not show a significant triple interaction ‘Condition’ X ‘TMS administration’ X ‘Muscle’ interaction ($F_{4.54,154.49} = .3$, $p = .898$, $\eta_p^2 < .01$). Indeed, only a significant main effect of factor ‘TMS administration’ ($F_{2.28,77.43} = 5.02$, $p = .007$, $\eta_p^2 = .13$) and ‘Muscle’ ($F_{1,34} = 54.97$, $p < .001$, $\eta_p^2 = .62$) were found. Again, the factor ‘Condition’ did not reach statistical significance ($F_{2,68} = .63$, $p = .537$, $\eta_p^2 = .02$) and planned comparisons conducted for ‘static hand’ trials showed that ($\log_{10}$) MEP amplitudes did not differ among grasping conditions (for ECR: intransitive *vs.* object: $t_{34} = -.56$; $p = .577$; $d = .1$; intransitive *vs.* social: $t_{34} = .09$; $p = .931$; $d = .01$; object *vs.* social: $t_{34} = .76$; $p = .454$; $d = .13$; for FCR: intransitive *vs.* object: $t_{34} = -.99$; $p = .331$; $d = .17$; intransitive *vs.* social: $t_{34} = -1.93$; $p = .061$; $d = .33$; object *vs.* social: $t_{35} = -1.57$; $p = .126$; $d = .27$; **Supplemental Figure 1b**). No other significant effect was found (all *Fs* < 2.35, all *ps* > .103).

Both these analyses corroborate the pattern of results found exploiting the motor resonance index and reported as main analyses in our work, ruling out a general facilitatory effect of social stimuli.

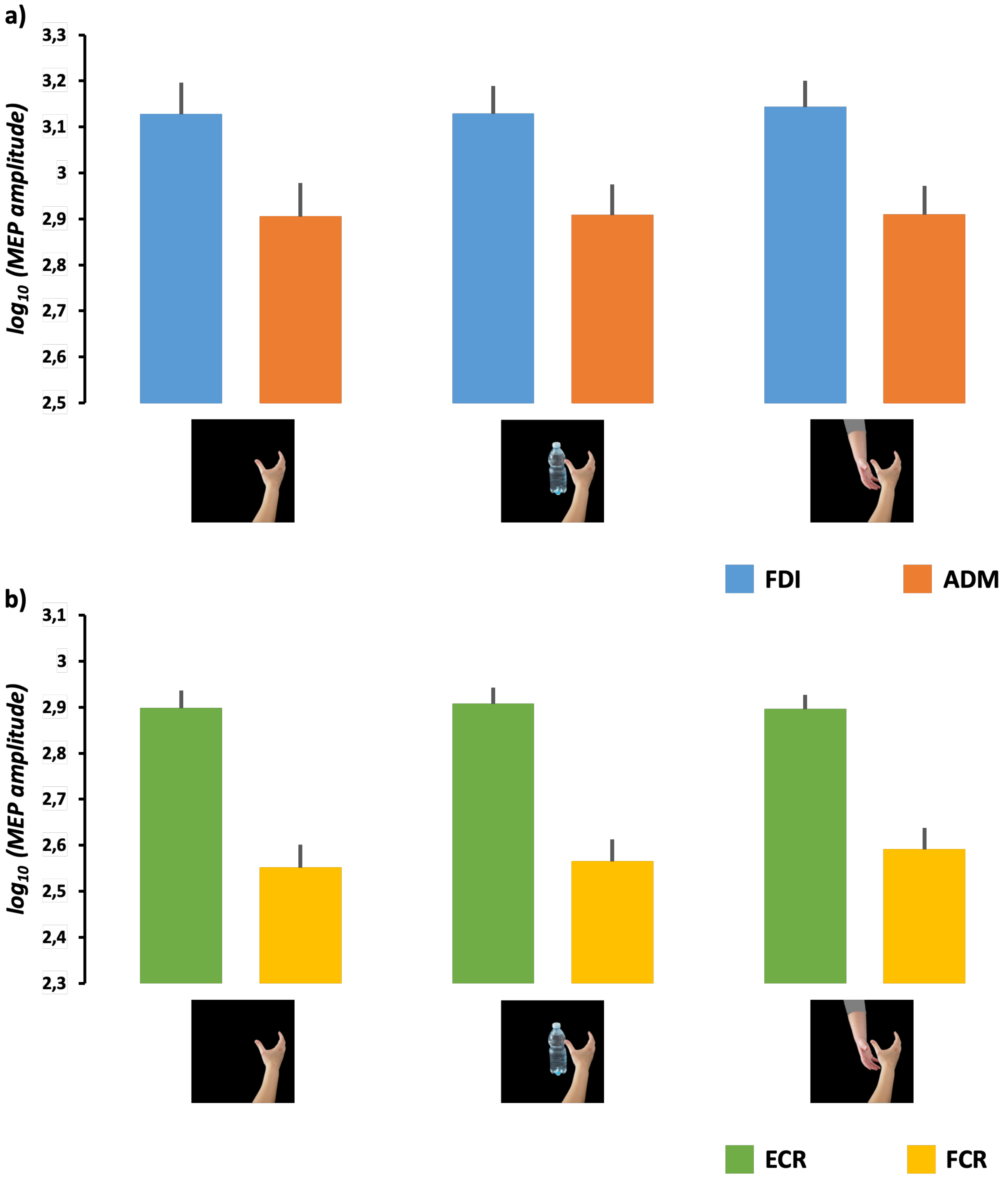

Supplemental Figure 1. Raw MEPs values during the observation of 'static hand' trials in the three experimental conditions ('intransitive grasping'; 'object grasping'; 'social grasping') and for the four muscles (**a**: FDI – straight blue lines, ADM – dotted brown lines; **b**: ECR – straight green lines, FCR – dotted yellow lines). No statistically significant differences in corticospinal excitability were found. Error bars: SE.
